# Resistant Starch Attenuates Bone Loss in Ovariectomised Mice by Regulating the Intestinal Microbiota and Bone-Marrow Inflammation

**DOI:** 10.3390/nu11020297

**Published:** 2019-01-30

**Authors:** Yuko Tousen, Yu Matsumoto, Yuya Nagahata, Isao Kobayashi, Masahiro Inoue, Yoshiko Ishimi

**Affiliations:** 1 Department of Food Function and Labeling, National Institute of Health and Nutrition, National Institutes of Biomedical Innovation, Health and Nutrition, 1-23-1 Toyama, Shinjuku-ku, Tokyo 162-8636, Japan; tousen@nibiohn.go.jp (Y.T.); yumatsu@kanto-gakuin.ac.jp (Y.M.); 2 Product Development Laboratory, J-OIL MILLs Inc., 11 Kagetoricho, Totsuka-ku, Yokohama, Kanagawa 245-0064, Japan; yuya.nagahata@j-oil.com (Y.N.); isao.kobayashi@j-oil.com (I.K.); masahiro.inoue@j-oil.com (M.I.)

**Keywords:** resistant starch, bone, osteoporosis, intestinal microbiota, inflammation

## Abstract

The intestinal microbiota may regulate bone metabolism by reducing levels of pro-inflammatory cytokines, and T cells in bone tissues of oestrogen-deficient mice have been reported. Resistant starch (RS) is a type of dietary fibre and results in changes in the composition of the gut microbiota. We evaluated the effects of RS supplemented in diets on intestinal microbial composition, bone mineral density, and inflammatory-gene expression in the colon and bone marrow of ovariectomised (OVX) mice. OVX mice were divided randomly into three groups: OVX control, OVX fed a 20% high amylose corn starch (HAS) diet, and OVX fed a 20% acid-hydrolysed HAS (AH-HAS) diet. HAS and AH-HAS diets contained 6.8% and 12% of RS, respectively. After 6 weeks, treatment with HAS or AH-HAS increased the abundance of *Bifidobacterium* spp. in faeces. The AH-HAS diet tended to upregulate mRNA expression of interleukin (IL)-10 in the colon, and downregulate expression of receptor activator of nuclear factor kappa-B ligand and IL-7 receptor genes in the bone marrow of OVX mice. AH-HAS treatment attenuated ovariectomy-induced bone loss. These findings suggest that AH-HAS might change the microbiota and immune status of the bone marrow, resulting in attenuated bone resorption in OVX mice.

## 1. Introduction

Osteoporosis is a skeletal disease that leads to an increased risk of fracture. Menopausal osteoporosis is a disorder involving high turnover of bone and bone loss attributed to oestrogen deficiency in women, and is a serious public-health problem worldwide [1]. Medical treatments for osteoporosis, such as hormone replacement and bisphosphonates, have undesirable side effects [2,3]. Therefore, preventive and therapeutic treatments for osteoporosis with fewer undesirable side effects are required.

Declining levels of oestrogen result in stimulation of bone resorption and, to a lesser extent, bone formation, thereby leading to rapid loss of bone [4,5]. This phenomenon has been suggested to be due to loss of the immunosuppressive effects of oestrogen (which results in increased production of the cytokines that promote osteoclastogenesis) and the direct effects of oestrogen on osteoclasts [4,6]. Osteoclastic bone resorption is driven by proinflammatory cytokines such as receptor activator of nuclear factor kappa-B ligand (RANKL) and tumour necrosis factor (TNF)-α produced by immune cells (e.g., activated T cells and B cells) [7,8].

In addition, increased permeability allows an expanded range of molecules and potential antigenic load to enter epithelial submucosa, which may initiate abnormal intestinal and systemic proinflammatory responses [9,10]. This process is critical in the context of bone homeostasis because osteoclastogenic cytokines are also produced by immune cells present in intestinal subepithelial compartments of the intestine, and any change in intestinal permeability is likely to increase levels of osteoclastogenic cytokines and affect bone density. Li et al., reported that a deficiency of sex steroids increases gut permeability, expands the number of T-helper-17 cells, and upregulates expression of the osteoclastogenic cytokines TNF-α, RANKL, and interleukin (IL)-17 in the small intestine and bone marrow [11].

The intestinal microbiota has a critical role in human health, and dysbiosis can result in inflammatory bowel disease, diabetes mellitus, or obesity [12,13]. This effect is due (at least in part) to the key role of the intestinal microbiota in shaping the immune system [12,13]. Recently, some studies have suggested an important role for gut–bone signalling pathways and the microbiota in modulating bone health through modification of immune status [14,15]. However, the mechanisms by which the gut and the intestinal microbiota modulate bone density are not understood completely.

Resistant starches (RSs) are a subgroup of dietary fibre that includes all starch and starch-degradation products that are not absorbed and digested in the small intestine of healthy individuals [16]. Though resistant to digestion in the small intestine, RSs can be used as substrates by bacterial species that reside in the colon, and these fermentations lead to an increase in levels of short-chain fatty acids (SCFAs) [16]. Several studies have characterised the capacity of RSs to induce alterations in the composition of the intestinal microbiota, including increases in *Bifidobacterium*, *Lactobacillus*, and *Bacteroides* spp. [17]. Moreover, it has been suggested that SCFAs generated from prebiotics regulate the number and function of regulatory T cells in the colon, thereby controlling inflammation [18]. Previously, we demonstrated that a combination of 9% RS and isoflavones inhibited oestrogen deficiency-induced bone loss in ovariectomised (OVX) mice, and that this combination has probiotic effects that increase the abundance of *Bifidobacterium* spp. in the intestinal microbiota of mice [19].

We hypothesised that RS intake would modulate the intestinal microbiota, as well as inflammation in the intestine, resulting in reduced inflammation of bone marrow and bone loss caused by oestrogen deficiency. Here, we evaluated the effect of 12% RS supplemented in a diet on the intestinal microbiota, bone mineral density (BMD), and expression of inflammatory and the genes of tight-junction proteins in the colon and bone marrow of OVX mice.

## 2. Materials and Methods

### 2.1. Animals and Diet

Female mice (ddY strain, 8 weeks) were purchased from Shizuoka Laboratory Animal Center (Shizuoka, Japan). Mice were housed in individual cages in a temperature- and humidity-controlled room (23 °C ± 1 °C and relative humidity of 60 ± 5%) with a 12-h light–dark cycle. Mice were given free access to an AIN-93G diet for 4 days before surgery was undertaken [20]. The mice were sham-operated (Sham group, *n* = 8) or underwent ovariectomy (OVX) on the same day. OVX mice were divided randomly into three groups (*n* = 8 each): OVX control (OVX), OVX fed a 20% high amylose corn starch (HAS)-supplemented diet (OVX + HAS), and OVX fed a 20% acid-hydrolysed high amylose corn starch (AH-HAS)-supplemented diet (OVX + AH-HAS). Mice were pair-fed their respective diets for 42 days with free access to distilled water during this period. AH-HAS contains higher amounts of RS than HAS; the hydrolysed granules have more crystalline regions and, thus, greater resistance to enzymatic digestion [21]. The concentrations of HAS and AH-HAS were determined in our previous studies; the 20% AH-HAS (12% RS) diet inhibited bone loss slightly in OVX mice [22]. The experimental diets were prepared according to the AIN-93G formulation (Table S1) [20]. Dry powdered HAS or AH-HAS, as indicated, were added to the diets instead of corn starch. In the supplemented diets, RSs were included as 40.5% and 68.0% of the dry weight for HAS and AH-HAS, respectively (Amylofiber^®^SH and HS-7, respectively; J-OIL MILLs, Tokyo, Japan) [21]. When preparing the diets, the RS content of HAS and AH-HAS was 34.4% and 60.0%, respectively (wet weight). HAS and AH-HAS diets contained 6.8% and 12% of RS, respectively. HAS and AH-HAS were kindly provided by J-OIL MILLs.

After 14 days of treatment, mice (all groups, *n* = 8 each) were euthanised by exsanguination under anaesthesia. The right tibia was removed to extract total RNA from bone-marrow cells. The proximal and distal sections of tibia were cut off, and an injection needle was stick into the bone marrow on the proximal and distal sections, respectively. Isogen^®^ II (Nippon Gene, Tokyo, Japan) solution was injected into the tibial bone marrow from the proximal and distal sections and the bone marrow flowed out with Isogen^®^ II from tibia. Isogen^®^ II was mixed with the bone marrow, and then stored at −80 °C until assay. After 40 days of treatment, faeces and 48-h urine were collected. Faeces placed immediately on dry ice for freezing, and stored at −80°C until assay, and 48-h urine were stored at −20°C until assay.

Mice (all groups, *n* = 8 each) were fasted overnight immediately before anatomical investigations. After 42 days of treatment, mice were euthanised by exsanguination under anaesthesia. The uterus was removed and its wet weight recorded. The caecum was removed with its contents, weighed, placed immediately on dry ice, and stored at −80 °C until assay. The left femur was removed, submerged in 70% ethanol, and stored at 4 °C until measurement of BMD. The right tibia was removed to extract total RNA from bone-marrow cells. A segment of the large intestine was collected from each mouse and its contents removed. Then, the tissue was washed gently with phosphate-buffered saline, submerged completely in RNAlater^®^ (Qiagen, Hilden, Germany) and stored at −80 °C until assay.

All procedures involving animals were in accordance with the *Guidelines for the care and use of laboratory animals* by the National Institute of Biomedical Innovation, Health and Nutrition (Tokyo, Japan) and the ethics committee approved the study protocol (DS27-61).

### 2.2. Radiographic Analyses of the Femur

BMD of the femur was quantified by dual-energy X-ray absorptiometry with a specialized software program for small animals (DCS-600EX-RIII, Aloka, Tokyo, Japan). Before measurement of the femur, calibration was performed using a phantom; all femoral bones were then scanned together. BMD was calculated using the bone mineral content of the measured area. Intra-assay and inter-assay coefficients of variation were less than 1.0% and 4.8%, respectively. The detection limit of BMD was 15 mg/cm^2^. The scanned area of the mouse femur was divided into three equal parts: proximal, midshaft, and distal.

### 2.3. Microcomputed Tomography (μCT) Analysis of the Distal Femur

Distal femurs were scanned at 48-μm intervals using an experimental animal CT system (LaTheta LCT-200; Hitachi Aloka Medical, Tokyo, Japan). Analyses of distal femurs were performed in a region of the trabecular and cortical bone to the growth plate extending 1.4 mm towards the diaphysis.

### 2.4. Weight of Caecal Content, pH, and β-Glucosidase Activity

Caecal content was collected, and caecal tissue washed with physiological (0.9%) saline and wiped. The weight of caecal content was calculated by subtracting the tissue weight from the total weight. The pH of caecal content was measured with a pH meter (B-212 Twin Compact; Horiba, Kyoto, Japan). Beta-glucosidase activity was measured by determining the amount of *p*-nitrophenol generated from *p*-nitrophenyl β-pyranoside. The reaction mixture contained 40 µL of substrate solution (50 mM phosphate buffer at pH 7.0 with 1 mM *p*-nitrophenyl β-pyranoside) and 10 µL of sample solution at a 1:9 (*w*/*w*) dilution of the caecal sample in 50 mM phosphate buffer at pH 7.0. This reaction mixture was incubated for 60 min at 37 °C, the *p*-nitrophenol concentration was measured at 405 nm by spectrophotometry after the addition of 200 µL of 0.1 M NaOH. Enzyme activity was expressed as moles of *p*-nitrophenol per total caecal content in 60 min.

### 2.5. Biochemical Marker of Bone Resorption

The bone resorption marker C-terminal telopeptide of type I collagen (CTX-I) in urine was measured by ELISA kits (RatLaps™ ELISA: Immunodiagnostic Systems, Boldon, UK), according to the manufacturer’s instructions.

### 2.6. DNA Extraction from Faeces

Bacterial DNA was extracted from faecal samples according to a method used by Nagashima et al., with modifications [23,24]. DNA was extracted from the suspension using a GC Series Genomic DNA Whole Blood kit (Wako Pure Chemical Industries, Osaka, Japan) and then purified using a Magtration^®^ system (12GC; Precision System Science, Chiba, Japan).

### 2.7. Polymerase Chain Reaction (PCR) Conditions and Terminal Restriction Fragment Length Polymorphism (T-RFLP) Analyses

Amplification of faecal 16S rDNA, digestion using restriction enzymes, size fractionation of terminal restriction fragments, and analyses of T-RFLP data were done according to the method used by Nagashima et al., with modifications [23,24]. Briefly, PCR was carried out using total faecal DNA with primers for 5′-carboxy-fluorescein-labelled 516f and 1510r. The resulting 16S rDNA amplicons were treated for 3 h at 55 °C with 10 U of Bs/I (5′-CCNNNNN|NNGG-3′) (New England Biolabs, MA, USA). The fluorescently labelled terminal restriction fragments produced by digestion with Bs/I were analysed by electrophoresis on an ABI PRISM 3130xl Genetic Analyser (Applied Biosystems, CA, USA) in GeneScan mode (injection time was 30 s, and run time was 40 min).

### 2.8. Analyses of Clone Libraries

Analyses of clone libraries were done according to the method used by Nagashima et al. [23] with modifications. Briefly, the fecal 16S rDNA clone libraries were constructed using the TOPO TA Cloning Kit (Thermo Fisher Scientific, MA, USA) and the sequences of both strands of the cloned NDA (E. coli positions 516 to 1510) were determined using the BigDye Terminator Cycle Sequencing Kit (Thermo Fisher Scientific, MA, USA). The sequence data were subjected to homology searches with the BLAST or FASTA program, the phylogenetic tree was constructed with the CLUSTAL W program and TreeView software (these programs are at the DDBJ web site, http://www.ddbj.nig.ac.jp/searches-e.html), and in silico restriction enzyme digestion was analysed with the Mac Vector software (Oxford Molecular Ltd., Oxford, UK). The sequence data for individuals C and G, and reference strains used for phylogenetic analysis methods are used by Nagashima et al. [23,24]. The length and peak areas of terminal restriction fragments were determined, and a phylogenetic tree constructed using GeneMaths (Applied Maths, Sint-Martens-Latem, Belgium). The fingerprint profiles generated by T-RFLP were compared using the Pearson correlation coefficient. The Dice Similarity Matrix was used to construct a dendrogram of T-RFLP fingerprint profiles. DNA extraction from faeces, analyses of TRFLP and clone libraries were conducted by TechnoSuruga Laboratory Co., Ltd. (Shizuoka, Japan).

### 2.9. RNA Extraction and Quantitative Real-Time PCR of Large-Intestine Tissue or the Bone Marrow of the Tibia

Total RNA was extracted from the bone marrow of the tibia using Isogen^®^ II according to manufacturer (Nippon Gene) instructions. Total RNA was extracted from large-intestine tissue using an RNeasy Lipid Tissue Mini kit according to manufacturer (Qiagen) directions.

Complementary DNA (cDNA) was synthesised from 1 μg of total RNA using PrimeScript™ RT Master Mix (TaKaRa Bio, Shiga, Japan). cDNA was quantified by real-time PCR using SYBR™ Premix Ex Taq II (TaKaRa Bio). PCR conditions were 95 °C for 30 s, followed by 40 cycles of 95 °C for 5 s and 60 °C for 30 s. The primer sequences are shown in Table S2. The results from bone-marrow cells are expressed as the fold-change relative to those of sham mice after normalisation to expression of the beta-actin (*Actb*) gene. The results from the intestine are expressed as the fold-change relative to those of sham mice after normalisation to expression of the 36B4 (*Rplp0*) gene.

### 2.10. Statistical Analyses

Data are the mean ± standard error of the mean (SEM). The influences of HAS or AH-HAS treatment upon the composition of the faecal intestinal microbiota were evaluated by analysis of variance (ANOVA) and Tukey’s post hoc test if the data had a normal distribution. If the data did not have a normal distribution and their variances were not equivalent, a non-parametric Kruskal–Wallis test was carried out to determine significant differences between groups. The statistical significance of differences in femoral BMD was determined by analysis of covariance and Bonferroni significant difference test. Body weight was used as a covariate in the analyses (ANCOVA) of femoral BMD to adjust for possible confounding effects. The remaining data were analysed using one-way ANOVA. Differences between groups were assessed by Tukey’s post hoc test. Differences were considered significant if *p* < 0.05. Statistical analyses were undertaken using SPSS v19 (IBM, Armonk, NY, USA).

## 3. Results

### 3.1. Body Weight and Tissue Weight

Significant differences in initial body weight, final body weight, or total food intake were not observed among groups (Table 1). The uterine weight from all OVX groups was significantly lower than that in the sham group. Treatment with HAS or AH-HAS did not affect uterine weight in OVX mice.

### 3.2. Weight of Caecal Content, pH, and β-Glucosidase Activity

The wet weight of caecal content was significantly higher in the OVX + AH-HAS group than that in the sham and OVX groups, and the wet weight of caecal content in the OVX + HAS group was significantly higher than that of the OVX group (Table 1). The caecal pH was significantly lower in the OVX + AH-HAS group than that in the other groups (Table 1). The caecal pH in the OVX + HAS group was significantly lower than that in the OVX group. The caecal β-glucosidase activity was significantly higher in the OVX + AH-HAS group than that in the other groups (Table 1). These results suggested that caecal content, pH, and β-glucosidase activity were affected significantly by AH-HAS intake.

### 3.3. Biochemical Marker of Bone Resorption

Urinary CTX-I, a marker for bone resorption, was higher in the OVX, OVX + HAS and OVX + AH-HAS groups than that in sham group (Figure S2). There were no significant differences among the OVX groups.

### 3.4. Analyses of Faecal Microbiota

**Figure 1:**
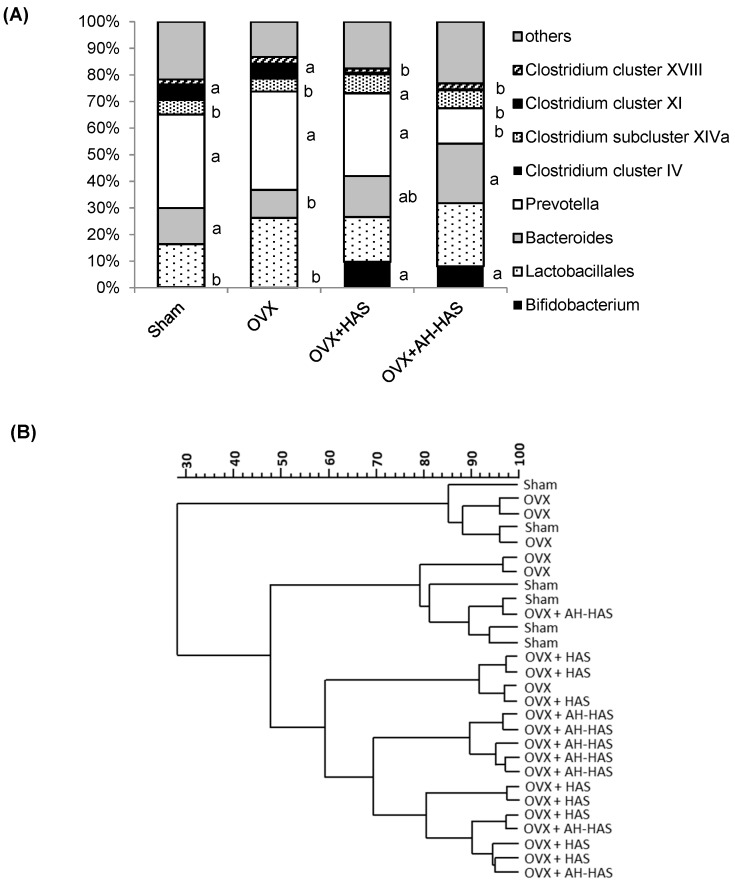
(**A**) Composition of caecal faecal microbiota in mice. Sham: sham-operated group; OVX: ovariectomised group; OVX + HAS: OVX + high-amylose corn starch diet group; OVX + AH-HAS: OVX + acid-hydrolysed high-amylose corn starch diet group. Values are the mean (sham and OVX groups: *n* = 6, OVX + HAS and OVX + AH-HAS groups: *n* =8), with their standard errors represented by vertical bars. Differences were considered significant if *p* < 0.05. The influences of HAS or AH-HAS treatments upon composition of the faecal intestinal microbiota were evaluated by ANOVA and Tukey’s *post hoc* test if the data had a normal distribution. If the data did not have a normal distribution and their variances were not equivalent, a non-parametric Kruskal–Wallis test was carried out to determine significant differences between groups. Differences were considered significant if *p* < 0.05. ^a, b^ Significant differences in the abundance of *Bifidobacterium* spp., *Bacteroides* spp., *Prevotella* spp., *Clostridium cluster IV* spp., *Clostridium cluster XI* spp. among the groups. (**B**) Cluster analysis of intestinal microbiota based on terminal restriction fragment length polymorphism (T-RFLP) analyses. T-RFLP was compared using Pearson’s correlation coefficient. The Dice Similarity Matrix was used to construct a dendrogram of the T-RFLP fingerprint profiles.

The faecal microbiota of mice was influenced by intake of HAS or AH-HAS (Figure 1A,B). The abundance of *Bifidobacterium* spp. in the OVX + HAS and OVX + AH-HAS groups (9.75 ± 2.57% and 8.08 ± 2.07%, respectively) was significantly higher than that in the sham or OVX groups (0.22 ± 0.22% or 0.00 ± 0.00%, respectively) (Figure 1A). The abundance of *Bacteroides* spp. in the OVX + AH-HAS group (22.41 ± 2.01%) was significantly higher than that in the OVX group (10.54 ± 1.90%). The abundance of *Prevotella* spp. in the OVX + AH-HAS group (13.32 ± 3.77%) was significantly lower than those in the sham, OVX, or OVX + HAS groups (35.17 ± 4.97%, 36.96 ± 4.78% and 30.98 ± 1.94%, respectively). The abundance of *Clostridium cluster IV* spp. in the OVX + HAS and OVX + AH-HAS groups was 0.25 ± 0.04% and 0.04 ± 0.04%, respectively, but those species were not detected in the sham or OVX groups (Figure 1A). The abundance of *Clostridium cluster XI* in the OVX + HAS and OVX + AH-HAS groups (0.64 ± 0.41% and 0.35 ± 0.10%, respectively) was significantly lower than those in the sham and OVX groups (5.91 ± 1.04% and 5.61 ± 1.43%, respectively) (Figure 1A).

We undertook a cluster analysis of the intestinal microflora based on T-RFLP analyses (Figure 1B). The dendrogram was separated based on the intake of HAS or AH-HAS: one cluster with 60% similarity for mice in the groups HAS or AH-HAS with an exception (one individual OVX in the cluster).

### 3.5. Expression of Genes Associated with Inflammation or Tight-Junction Proteins in the Colon

**Figure 2:**
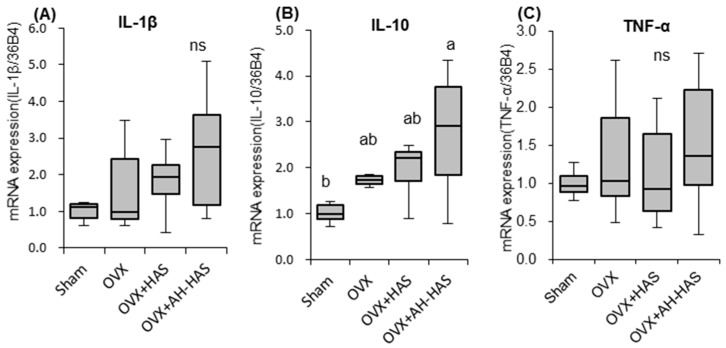
mRNA expression of IL-1β (**A**), IL-10 (**B**) and TNF-α (**C**) in the colons of mice. mRNA of the colon was obtained from sham mice and OVX mice fed a control diet, 20% high-amylose corn starch (HAS)-supplemented diet (OVX + HAS), or an 20% acid-hydrolysed high-amylose corn starch (AH-HAS)-supplemented diet (OVX + AH-HAS) for 42 days. mRNA expression was determined by qRT-PCR. The ordinate axis indicates the relative amount of mRNA compared with that in sham mice. Gene expression was expressed as the fold-change relative to sham mice and normalised with 36B4. Values are the mean ± SEM (*n* = 8). mRNA expression in the colon was assessed using one-way analysis of variance (ANOVA). Differences between treatment groups were assessed by Tukey’s *post hoc* test. Differences were considered significant if *p* < 0.05. ^a, b^ Mean values with unlike letters are significantly different and ns: not-significant.

Figure 2 shows expression of inflammation-related genes in mice colons. mRNA expression of IL-10 in the OVX + AH-HAS group was significantly higher than that in the sham group (Figure 2B). mRNA expression of IL-1β tended to be higher in the OVX + AH-HAS group compared with that in the sham group (*p* = 0.057) (Figure 2A). There was no significant difference in the gene expression of TNF-α among the groups (Figure 2C). There were no significant differences in the gene expression of tight-junction proteins among the groups (Supplement Figure S1).

### 3.6. Expression of Inflammation-Related Genes in Bone Marrow

**Figure 3:**
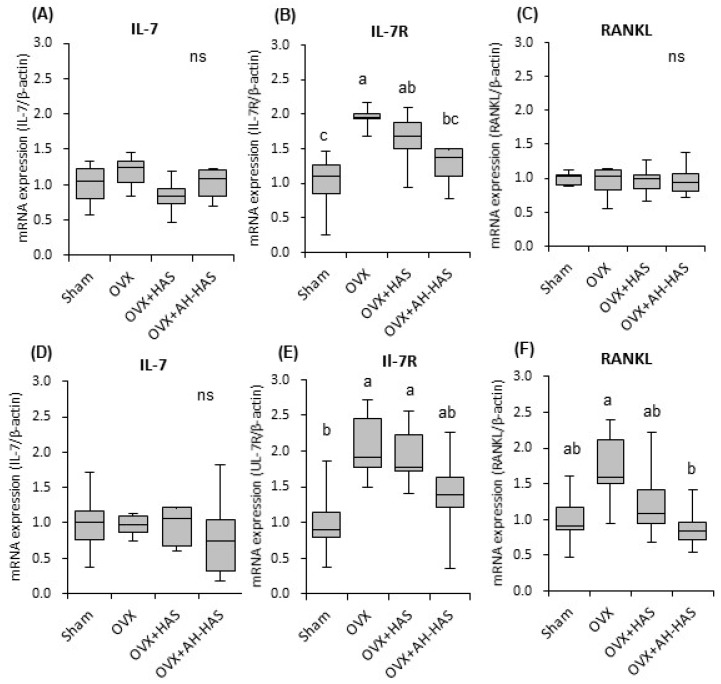
mRNA expression of bone-marrow cells collected from the tibia was measured from sham and OVX mice fed a control diet, 20% high-amylose corn starch (HAS)-supplemented diet (OVX + HAS), or 20% acid-hydrolysed high-amylose corn starch (AH-HAS)-supplemented diet (OVX + AH-HAS) for 14 (**A–C**) or 42 (**D**–**F**) days. Expression of IL-7 (**A**,**D**), IL-7R (**B**,**E**) and RANKL (**C**,**F**) were determined by qRT-PCR. The ordinate axis indicates the relative amount of mRNA compared with that in sham mice. Gene expression was expressed as the fold-change relative to that of sham mice and normalised to β-actin expression. Values are the mean ± SEM (*n* = 8). mRNA expression of bone-marrow cells collected from the tibia was measured using one-way analysis of variance. Differences between treatment groups were assessed by Tukey’s *post hoc* test. Differences were considered significant if *p* < 0.05. ^a, b, c^ Mean values with unlike letters are significantly different and ns: not-significant.

Expression of inflammation-related genes in the bone marrow of mice is shown in Figure 3. mRNA expression of IL-7R in OVX mice was significantly higher than that in sham mice on day-14 and day-42 (Figure 3B,E). On day-14, mRNA expression of IL-7R in the OVX + AH-HAS group was significantly lower than that in the OVX group (Figure 3B). On day-42, mRNA expression of IL-7R in the OVX + AH-HAS group tended to be lower than that in the OVX group (*p* = 0.090) (Figure 3E). There were no significant differences in mRNA expression of IL-7 among the groups at day-14 or day-42 (Figure 3A,D). There were no significant differences in mRNA expression of RANKL among the groups on day-14 (Figure 3C). On day-42, mRNA expression of RANKL in the OVX group tended to be higher than that in the sham group (*p* = 0.053) (Figure 3F). On day-42, mRNA expression of RANKL in the OVX + AH-HAS group was significantly lower than that in the OVX group (Figure 3F). There were no significant differences in the ratio of expression of RANKL/OPG among the groups on days 14 or 42 (data not shown). There were not significant effects of HAS or AH-HAS on the expressions of TNF-α and nuclear factor of activated T cells c1 (NFATc1) in bone marrow cells on days 14 or 42 (data not shown).

### 3.7. BMD of the Femur

**Figure 4:**
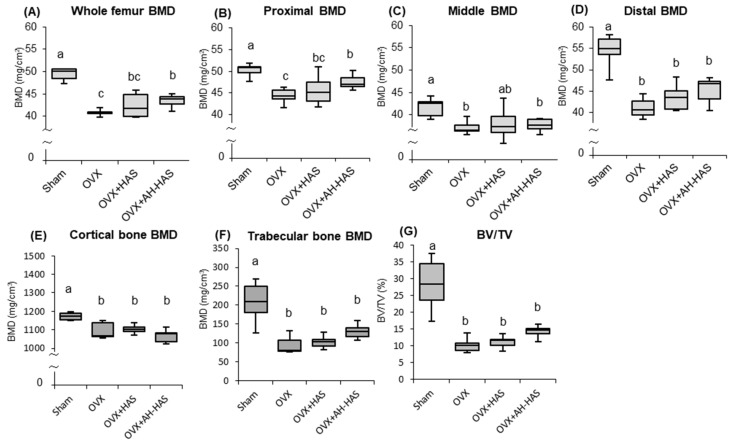
Bone mineral density (BMD) of the femur was obtained from sham and OVX mice fed a control diet, 20% high-amylose corn starch (HAS)-supplemented diet (OVX + HAS), or 20% acid-hydrolysed high-amylose corn starch (AH-HAS)-supplemented diet (OVX + AH-HAS) for 42 days. BMD of the (**A**) whole femur (**B**) proximal femur, (**C**) middle femur and (**D**) distal femur were analysed by DXA. BMD of the (**E**) Cortical and (**F**) trabecular bone, and (**G**) bone volume per tissue volume (BV/TV) on distal femur were analysed by uCT. Values are the mean ± SEM (*n* = 8). Significant differences in BMDs were determined by analysis of covariance (ANCOVA) and the Bonferroni significant difference test. Differences were considered significant if *p* < 0.05. Body weight was used as a covariate in the analysis of BMDs to adjust for a possible confounding effect. ^a, b, c^ Mean values with unlike letters are significantly different.

BMDs of the whole as well as the proximal, middle, and distal regions of the femurs of OVX mice were significantly lower than those of the sham group (Figure 4A–D). AH-HAS treatment attenuated bone loss in the whole and proximal region of the femur significantly (Figure 4A,B). There were no significant differences among the OVX groups with regard to the BMD of the middle and distal region of the femur (Figure 4C,D).

While the trabecular and cortical bone BMDs, and bone volume per tissue volume (BV/TV) of the distal femur were significantly lower in the OVX groups than those of the sham group, there were no significant differences among the OVX groups (Figure 4E–G). However, AH-HAS treatment significantly attenuated the trabecular bone loss and the reduction in BV/TV, when the statistical analysis in trabecular bone BMD were performed by ANCOVA among the OVX, OVX+HAS, and OVX + AH-HAS groups (data not shown).

## 4. Discussion

We evaluated the effects of RS supplemented in diets on the intestinal microbial composition, BMD, and expression of inflammation-associated genes in the colon and bone marrow of OVX mice. We found that treatment with HAS or AH-HAS increased the abundance of *Bifidobacterium* spp. in mice faeces. AH-HAS downregulated expression of the osteoclastogenic cytokine RANKL and inflammation-related IL-7R genes in the bone marrow. AH-HAS treatment attenuated ovariectomy-induced bone loss. We demonstrated, for the first time, that the alleviative effect of RS feeding on bone loss was associated with inhibition of expression of ovariectomy-induced inflammatory cytokines and osteoclastic mRNA in bone-marrow cells.

Our results showed that AH-HAS had greater effects than HAS on the weight of caecal content, pH, β-glucosidase activity, changes in the composition/diversity of the microbiota, and gene expression of anti-inflammatory cytokines in the colon (Table 1, Figure 1A,B, Figure 2). In healthy individuals, starch entering the large bowel is metabolised actively by the numerous saccharolytic bacteria inhabiting that region of the intestine, thereby resulting in SCFA production and a concomitant reduction in luminal pH [16]. Acidification of the intracolonic environment is considered important for suppression of the production and activity of a range of bacterial metabolites implicated in colonic disease. Be-ta-glucosidase, an enzyme generated by the intestinal microbiota, contributes to the hydrolysis of glucose monomers from the non-starch polysaccharides. Thus, the increase in β-glucosidase activity might exhibit the change in microbiota composition/diversity.

The selectively of dietary fibre regulates intestinal bacteria (at least in part) by stimulating the growth of health-promoting bacterial species (*Bifidobacterium* and *Lactobacillus*) [25,26]. Those observations are agreement with the findings of our study: HAS and AH-HAS increased the abundance of *Bifidobacterium* spp. (Figure 1A). In a previous study, it was reported that mice fed on diets containing 18 and 36% RS were colonized with higher levels of *Bacteroidetes* and *Bifidobacterium*, *Akkermansia*, and *Allobaculum* species in proportions that were dependent on the concentration of the RS [27]. According to Hu et al., an RS diet caused shifts in microbial composition/diversity, including increase in the abundance of *Bifidobacterium* spp., increased production of SCFAs, and reduced inflammation [28]. Conversely, in the present study, AH-HAS decreased the abundance of *Clostridium XI* spp., but there were no effects on clusters of *Clostridum IV* or *XIVa*. It has been reported that *Clostridium* spp. can promote increased expression of IL-10 in regulatory T cells [29]. Whether the decrease/increase in *Clostrida* spp. can cause inflammation is not known, but it has been speculated that they are related to immune homeostasis and inflammation.

In the colon, we assessed mRNA expression of the proinflammatory cytokines TNF-α and IL-1β, the anti-inflammatory cytokine IL-10, and tight-junction proteins. mRNA expression of IL-10 was significantly higher in the AH-HAS group than that in the sham group. IL-10 is an essential component of the immune system within the intestinal tract, where it dampens inflammation and helps to restore tissue homeostasis. Bassaganya-Riera et al. reported that dietary supplementation with RS decreased ileal and colonic inflammatory lesions, and increased IL-10 expression in the splenocytes of wild-type mice [30]. In addition, tight-junction proteins play crucial parts in maintaining the integrity of the intestinal barrier. According to Vaziri et al., consumption of a RS-supplemented diet significantly increased intestinal expression of tight-junction genes, claudin-1 and occludin in rats [31]. However, we did not find significant effects of oestrogen deficiency or diets supplemented with HAS or AH-HAS on expression of TNF-α, IL-1β (Figure 2A,C) or tight-junction proteins in the colon (Supplemental Figure S1). Further studies (including on intestinal immune cells) are required to examine the direct effects of RSs on intestinal inflammation and tight junctions.

In the present study, AH-HAS treatment has been found to attenuate bone loss in the whole and proximal region of the femur significantly (Figure 4A,B). There were no statistically significant effects of AH-HAS in BMD of the trabecular and cortical bones, although AH-HAS treatment tended to attenuate trabecular bone loss, and the reduction in BV/TV. The results of femoral BMD analyses were not consistent between those measured by DXA and µCT. Whole femoral BMDs were measured by DXA analysis, whereas BMDs measured by μCT analysis were partial sections of trabecular and cortical bone in the distal femur. Additionally, DXA analysis was performed using the bone mineral content of the measured area, which is a two-dimensional analysis; conversely, μCT analysis is a three-dimensional analysis. Thus, we speculate that differences between these data were caused by variations arising from the analytical sections, as well as differences in the principle of femur measurement.

Oestrogen deficiency leads to an increase in bone resorption through increased osteoclastogenesis and osteoclast activity, resulting in the progression of osteoporosis [4,6,32]. Our current study indicated that oestrogen deficiency decreased the femoral BMD of OVX mice; HA-HAS attenuated bone loss in the OVX mice. However, there was no effect of HAS or AH-HAS treatment on urinary CTX-I in OVX mice. AH-HAS treatment attenuated OVX-induced bone loss moderately; hence, it might reduce the urinary CTX-I level if the AH-HAS treatment lasts for a longer duration.

Some researchers have reported that the effect of resistant starch increases calcium and magnesium absorption [33,34]. A potential mechanism for this effect is hypothesized to be the caecal wall hypertrophy and caecal acidification that RS-induced fermentation produces in the colon. There are a few reports on the effect of RS on BMD. Zafar et al., reported that diet treated with 5% RS did not increase calcium absorption capacity, and did not affect femoral BMD in male rats, which were fed with the recommended levels of dietary calcium (0.5%) [35]. Although the dietary calcium level used in the study was the same as our study, the diet treated with AH-HAS (12% RS) attenuated the OVX induced bone loss in our study. The effects of AH-HAS on BMD in sham mice were not examined in our study. In future, it is also necessary to examine the effect of AH-HAS on BMD in normal mice.

We hypothesized that RS intake would modulate the intestinal microbiota, resulting in reduced inflammation of bone marrow and bone loss caused by oestrogen deficiency. Therefore, we assessed the expression levels of cytokines related to inflammation and bone resorption in bone marrow cells. AH-HAS attenuated ovariectomy-induced bone loss (Figure 4A,B), and inhibited the ovariectomy-induced increases in expression of IL-7R mRNA on day-14 and day-42, and RANKL expression on day-42 (Figure 3B,E,F) in bone-marrow cells. The association between inflammation of the bone marrow and bone loss is well established. Studies have shown that bone inflammation is increased in the context of oestrogen deficiency and linked to osteoclastogenesis, and that bone resorption is derived by cytokine-producing activated T cells [8]. IL-7 regulates homeostasis of T cells and B cells via IL-7R signalling, and IL-7 causes bone resorption through activation of T cells and T cell-dependent augmentation of osteoclastogenesis [36]. There were no significant effects of AH-HAS on expression of IL7, but IL-7R suppressed the increase in OVX-induced IL-7R expression (Figure 3B,E). In our previous study, soy isoflavones and/or RS inhibited the increase in OVX-induced IL-7R mRNA expression [19]. Thus, these results suggested that AH-HAS might attenuate the bone loss caused by oestrogen deficiency via regulation of T cells.

RANKL is an important osteoclast-specific gene and a critical regulator of osteoclast development and bone metabolism [8]. RANKL expression is upregulated by inflammation related-cytokines and hormones. On day-14, we did not observe any significant effects of AH-HAS on expression of RANKL mRNA, although we did observe some effects on IL-7R expression in the bone marrow, as well as significant AH-HAS effects on RANKL expression on day-42. Therefore, these results suggest: (i) ovariectomy-induced expression of pro-inflammatory in bone-marrow cells; (ii) ovariectomy-induced osteoclastic RANKL expression might be downregulated by a diet supplemented with AH-HAS.

## 5. Conclusions

The present study demonstrated that OVX mice fed an AH-HAS-containing diet accelerated fermentation in the caecum, and increased the abundance of *Bifidobacterium* spp. in faeces. The AH-HAS diet tended to upregulate mRNA expression of the anti-inflammatory cytokine IL-10 in the colon, and downregulate expression of the osteoclastogenic cytokine RANKL and IL-7R genes in the bone marrow of OVX mice. AH-HAS treatment attenuated ovariectomy-induced bone loss.

These findings suggest that AH-HAS might change the microbiota and immune status of the bone marrow, resulting in attenuated bone resorption in OVX mice. Taken together, our findings might contribute to the evidence linking the intestinal microbiota, inflammation-related genes in the colon and bone marrow, and bone loss in OVX mice fed an AH-HAS diet.

## Figures and Tables

**Table 1:** Body weight, food intake, wet weight of caecal content, pH and β-glucosidase activity in mice on day 42.

	Sham	OVX	OVX + HAS	OVX + AH-HAS
Body weight				
Initial body weight (g)	28.1 ± 0.3	29.2 ± 0.4	28.1 ± 0.4	27.7 ± 0.4
Final body weight (g)	30.5 ± 1.5	33.7 ± 0.4	33.7 ± 0.8	32.5 ± 0.5
Total food intake (g)	173.7 ± 3.0	173.0 ± 2.7	173.4 ± 2.3	173.9 ± 2.3
Uterine weight (g)	77.6 ± 12.4 ^a^	13.8 ± 1.0 ^b^	15.0 ± 1.2 ^b^	17.0 ± 1.4 ^b^
Caecum				
Wet weight of caecal content (g)	0.214 ± 0.024 ^bc^	0.178 ± 0.033 ^c^	0.310 ± 0.030 ^ab^	0.409 ± 0.037 ^a^
Caecal content pH	7.850 ± 0.056 ^ab^	8.000 ± 0.037 ^a^	7.663 ± 0.073 ^b^	7.413 ± 0.079 ^c^
Caecal β-glucosidase activity *	1.099 ± 0.143 ^b^	1.341 ± 0.098 ^b^	1.873 ± 0.130 ^b^	4.057 ± 0.687 ^a^

^1^ Sham, sham-operated mice fed a control diet; OVX, ovariectomised mice fed a control diet; OVX + HAS, OVX mice fed a 20% high-amylose corn starch (HAS)-supplemented diet; OVX + AH-HAS, OVX mice fed a 20% acid-hydrolysed high-amylose corn starch (AH-HAS)-supplemented diet. Values are the mean ± SEM (*n* = 8). Differences were considered significant when *p* < 0.05. ^a, b, c^ Mean values with unlike letters were significantly different. * mol of *p*-nitro phenol/whole caecal content/60 min.

## Supplementary Materials

The following are available online at http://www.mdpi.com/2072-6643/11/2/297/s1: Figure S1: Gene expression of tight-junction proteins in the colon, Figure S2: Biochemical marker of bone resorption, Table S1: Composition of experimental diets, Table S2: Sequence of primers used for quantitative real-time PCR.
