# Supplementary material for: Resistant Starch Attenuates Bone Loss in Ovariectomised Mice by Regulating the Intestinal Microbiota and Bone-Marrow Inflammation
Source: Nutrients. 2019 Jan 30;11(2):297. doi: 10.3390/nu11020297 (PMC6412451; doi:10.3390/nu11020297)
Supplement: Supplementary file 1 [file nutrients-11-00297-s001.zip › Supplementary Files_final/RSÿ_ò╢_Tbles_S1-2_revised.docx]

**Table S1** Composition of the experimental diets.

(g/kg diet)^a^

|  | Ingredient | control ^b^ | 20% HAS diet ^c^ | 20% AH-HAS diet ^d^ |
| --- | --- | --- | --- | --- |
|  | Cornstarch | 529.5 | 329.5 | 329.5 |
|  | Casein | 200 | 200 | 200 |
|  | Sucrose | 100 | 100 | 100 |
|  | Corn oil | 70 | 70 | 70 |
|  | Cellulose | 50 | 50 | 50 |
|  | Mineral mixture ^a^ | 35 | 35 | 35 |
|  | Vitamin mixture ^a^ | 10 | 10 | 10 |
|  | L-Cystine | 3 | 3 | 3 |
|  | Choline bitartrate | 2.5 | 2.5 | 2.5 |
|  | Tert-Butylhydroquinone | 0.014 | 0.014 | 0.014 |
|  | High-amylose corn starch^e^ | - | 200 | - |
|  | Acid-hydrolysed  high-amylose corn starch^f^ | - | - | 200 |
|  | Total | 1000 | 1000 | 1000 |

^a^ Prepared according to the AIN-93G formulation [20].

^b^ Control diet.

^c^ High-amylose corn starch-supplemented diets.

^d^ Acid-hydrolysed high amylose corn starch-supplemented diets.

^e^ 40.5% resistant starch was included in high-amylose corn starch (HS-7; J-OIL MILLs). When prepared the diet, RS content of high amylose corn starch was 34.4 % (wet weight).

^f^ 68% resistant starch was included in acid-hydrolysed high-amylose corn starch (AH-HAS) (Amylofiber®SH; J-OIL MILLs). When prepared the diet, RS content of high amylose corn starch was 60% (wet weight).

HAS and AH-HAS diets contained 6.8% and 12% of RS, respectively.

**Table S2** Sequence of primers used for quantitative real-time PCR.

|  | Protein (gene) | Forward primer (5’ to 3’) | Reverse primer (5’ to 3’) |
| --- | --- | --- | --- |
|  | Colon |  |  |
|  | 36B4 | 5′-GGCCCTGCACTCTCGCTTTC-3′ | 5′-TGCCAGGACGCGCTTGT-3 |
|  | IL-1β ^a^ (*Il1b*) | 5′-TGCCACCTTTTGACAGTGAT-3 | 5′- CGAGATTTGAAGCTGGATGC-3′ |
|  | IL-10 ^b^ (*Il10*) | 5′-TAAGGGTTACTTGGGTTGCC-3′ | 5′-AAATCGATGACAGCGCCT-3′ |
|  | TNF-α ^c^ (*Tnf*) | 5′-ATGAGCACAGAAAGCATGATC-3′ | 5′-TACAGGCTTGTCACTCGAATT-3′ |
|  | Claudin-1 (*Cldn1*) | 5′-GATGTGGATGGCTGTCATTG-3′ | 5′-CCTGGCCAAATTCATACCTG-3′ |
|  | Claudin-3 (*Cldn3*) | 5′-ACTACCAGCAGTCGATGAAC-3′ | 5′-CTAGCAAGCAGACTGTGTGT-3′ |
|  | Claudin-15 (*Cld15*) | 5′-CTCTCATGATCACCGCCATC-3′ | 5′-CACAGGCTCCAGCAAGTATG-3′ |
|  | Occludin (*Ocln*) | 5′- CACACTTGCTTGGGACAGAG-3′ | 5′-TAGCCATAGCCTCCATAGCC-3′ |
|  | JAM-3 ^d^ (*Jam3*) | 5′-ACAAGATTCAAGGAGACCTGG-3′ | 5′-TTAGAGCAACGACCTCACAG-3′ |
|  | ZO-1 ^e^ (*Tjp1*) | 5′-AGGACACCAAAGCATGTGAG-3′ | 5′-GGCATTCCTGCTGGTTACA-3 |
|  | Bone marrow |  |  |
|  | β-actin (*Actb*) | 5′-CCACAGCTGAGAGGGAAATC-3′ | 5′-AAGGAAGGCTGGAAAAGAGC-3 |
|  | IL -7^f^ (*Il7r*) | 5′-TCCTCCACTGATCCTTGTTC-3′ | 5′-CTTCAACTTGCGAGCAGCAC-3′ |
|  | IL-7R ^g^ (*Il7r*) | 5′-GCGGACGATCACTCCTTCTG-3′ | 5′-AGCCCCACATATTTGAAATTCCA-3 |
|  | RANKL^h^ | 5'-TGAAGACACACTACCTGACTCCTG-3` | 5`-CCACAATGTGTTGCAGTTCC-3` |

^a^ Interleukin-1β, ^b^ Interleukin-10, ^c^ tumour necrosis factor α, ^d^ Junctional adhesion molecule, ^e^ Zonula occludens-1, ^f^ Interleukin-7, ^g^ Interleukin-7 receptor, ^h^ Receptor activator of nuclear factor kappa-B ligand
