# Supplementary figures and images for: Resistant Starch Attenuates Bone Loss in Ovariectomised Mice by Regulating the Intestinal Microbiota and Bone-Marrow Inflammation
Source: Nutrients. 2019 Jan 30;11(2):297. doi: 10.3390/nu11020297 (PMC6412451; doi:10.3390/nu11020297)

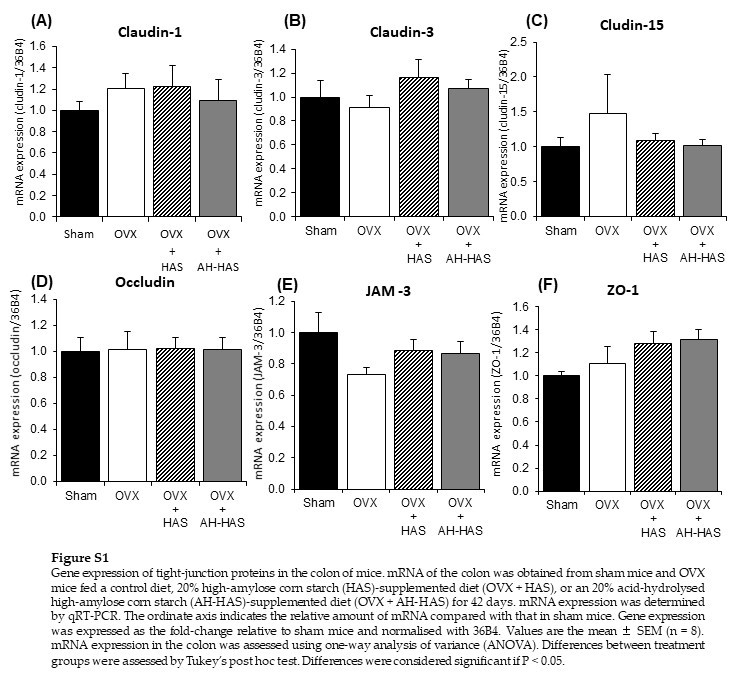

Supplement: Supplementary file 1 [file nutrients-11-00297-s001.zip › Supplementary Files_final/RSÿ_ò╢_Figure_S1.jpg]

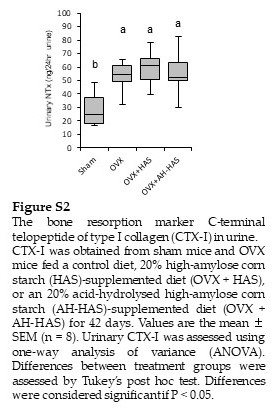

Supplement: Supplementary file 1 [file nutrients-11-00297-s001.zip › Supplementary Files_final/RSÿ_ò╢_R1_Figure_S2.jpg]
